# Real-Life Orthokeratology for Myopia Control in Hungarian Children: A Five-Year Study of Axial Length Changes

**DOI:** 10.3390/life16060929

**Published:** 2026-06-01

**Authors:** Beáta Tapasztó, János Németh, Illés Kovács, Zoltán Zsolt Nagy

**Affiliations:** 1 Department of Ophthalmology, Semmelweis University, Mária u. 39, H-1085 Budapest, Hungary; nemeth.janos@semmelweis.hu (J.N.); kovacs.illes@semmelweis.hu (I.K.); nagy.zoltan.zsolt@semmelweis.hu (Z.Z.N.); 2 Faculty of Health Sciences, Semmelweis University, Vas u. 17, H-1088 Budapest, Hungary

**Keywords:** myopia progression, myopia control, orthokeratology, axial length

## Abstract

**Purpose:** Orthokeratology is an established intervention for myopia control, but long-term European real-world data remain limited. This study evaluated 5-year axial length (AL) progression in Hungarian children undergoing orthokeratology and explored predictors of treatment response. **Methods:** This retrospective longitudinal study included 100 myopic children (198 eyes) treated with orthokeratology and followed for 5 years. AL was measured annually by optical biometry. By design, only children with uninterrupted follow-up on the same biometry device were included. Marginal multivariable regression with cluster-robust standard errors clustered on participant ID was used as the primary analytical approach; a linear mixed-effects model was applied as a sensitivity analysis. Responder analysis was performed using a biologically anchored threshold of <0.125 mm/year. **Results:** Mean cumulative AL elongation was 0.05 mm at 1 year and 0.34 mm at 5 years, corresponding to an annualised rate of 0.07 mm/year. First-year axial elongation was associated with 5-year AL change (r = 0.74, *p* < 0.001). Younger baseline age was the main predictor of greater AL progression, whereas baseline refractive status and sex showed no significant independent association with AL change. Responder rates were high, with 85.4% at eye level and 83.0% at the participant level. **Conclusions:** Orthokeratology was associated with low axial elongation over 5 years in this Hungarian paediatric cohort. These findings suggest favourable long-term axial length outcomes during orthokeratology treatment in routine clinical practice and underscore the importance of baseline age, whereas baseline refractive error did not show a significant independent association with treatment response.

## 1. Introduction

Myopia is the most common refractive disorder worldwide, with Holden et al. projecting that by 2050 approximately 4.76 billion people (49.8% of the world’s population) will be myopic and 938 million (9.8%) highly myopic [1]. In a recent European meta-analysis including 14 countries, Hungary had the second-highest reported myopia prevalence (43.24%) among the studied populations [2].

Myopia has become an increasingly important public health concern worldwide because of its rapidly rising prevalence and its association with a higher lifetime risk of potentially vision-threatening complications, particularly in eyes with excessive axial elongation [3,4]. Interventions aimed at slowing axial length (AL) growth in childhood are therefore of major clinical relevance, as AL is considered one of the most robust structural markers of myopia progression and treatment response [5,6,7]. In recent years, several therapeutic strategies have been introduced for myopia control, including low-dose atropine, specially designed spectacle lenses, soft contact lenses for myopia control, orthokeratology and light therapy [8,9].

Orthokeratology is one of the earliest and most widely used optical approaches for myopia control in children [10,11]. By temporarily reshaping the cornea through overnight wear of reverse-geometry rigid gas-permeable contact lenses, orthokeratology provides clear unaided daytime vision while also altering peripheral retinal defocus, a mechanism thought to contribute to the slowing of axial elongation [10,11]. Previous controlled studies, systematic reviews, and meta-analyses have shown that orthokeratology can reduce AL elongation in myopic children compared with single-vision spectacle correction, with reported treatment effects generally in the range of approximately 30% to 60%, although substantial inter-individual variability remains [11,12].

Despite this growing evidence base, several clinically relevant questions remain unresolved. The long-term axial length changes during orthokeratology appear to vary between individuals, and treatment response may be influenced by baseline age, refractive error, and other ocular or demographic factors [13,14]. More recent studies have specifically emphasised the importance of identifying predictors of treatment response, as some children exhibit very limited axial elongation during orthokeratology, whereas others continue to progress despite treatment [13,14,15,16,17]. In addition, while the short- and medium-term efficacy of orthokeratology has been demonstrated in multiple studies, comparatively fewer datasets provide continuous long-term follow-up with repeated biometric measurements extending beyond 3 years, particularly in European paediatric cohorts [18,19,20].

Orthokeratology was introduced at the Department of Ophthalmology, Semmelweis University, in 2005, initially mainly in adults as a non-surgical alternative to refractive laser surgery. Over time, the clinical focus of the department gradually shifted toward paediatric myopia control, in parallel with the expanding international evidence supporting the safety and efficacy of orthokeratology in children [21,22]. In Hungary, orthokeratology has also historically been requested for practical and sports-related reasons, particularly among children involved in aquatic sports, where spectacles or daytime contact lens wear may be inconvenient. This represents a contextual feature of local clinical demand and was not analysed as a predictor of axial elongation in the present study.

Over nearly two decades of clinical use, the orthokeratology program at this centre has expanded substantially, and nearly 700 orthokeratology treatments have been performed during this period. However, robust evaluation of long-term AL progression and treatment-associated biometric changes has been complicated using different measurement platforms over time, including earlier A-scan ultrasonography and later optical biometry devices such as the IOLMaster (Carl Zeiss, Jena, Germany) and Lenstar (Haag-Streit AG, Köniz, Switzerland). The COVID-19 pandemic also disrupted regular follow-up in a proportion of patients, further limiting the uniformity of available longitudinal data. For these reasons, the present study was designed as a retrospective cohort analysis of 100 paediatric patients selected based on consistent follow-up and a minimum observation period of 5 years using a uniform biometric approach within the analysed dataset.

The aim of the present study was to characterise long-term AL progression in children undergoing orthokeratology and to examine whether treatment-associated axial elongation remained stable over time. In addition, associations between AL progression and demographic and clinical variables were analysed, including age at treatment initiation, sex, prior optical correction and baseline refractive status, in order to identify potential predictors of treatment response.

The present study adds a real-world, single-centre Central-European cohort with 5 years of longitudinal follow-up and uniform axial length measurements obtained using the same optical biometry platform (Lenstar LS 900, Haag-Streit AG) throughout the analysed period. By combining a relatively large sample size (100 patients) with consistent biometric methodology, longitudinal analysis using bilateral eye data with appropriate correlation handling, and exploration of demographic and refractive predictors of axial elongation, this study aims to describe long-term axial length changes during orthokeratology in routine clinical practice. In addition, a biologically anchored responder definition based on the expected orthokeratology-related reduction in axial elongation reported in untreated myopic children was applied. We do not claim the longest European follow-up; rather, we add complementary real-world evidence to the prospective long-term datasets already published [18,20].

## 2. Materials and Methods

### 2.1. Study Design and Participants

This real-world longitudinal follow-up study analysed children who underwent orthokeratology fitting at the Department of Ophthalmology, Semmelweis University (Budapest, Hungary), between 2014 and 2020. Children were excluded if they had any ocular disease other than myopia, a history of ocular surgery, or any systemic or genetic condition known to be associated with myopia or abnormal axial elongation.

Inclusion criteria were: Caucasian ethnicity; regular attendance at follow-up visits throughout the observation period; availability of axial length measurements obtained with the same biometry device at all visits; and a minimum follow-up duration of 5 years.

**Figure 1:**
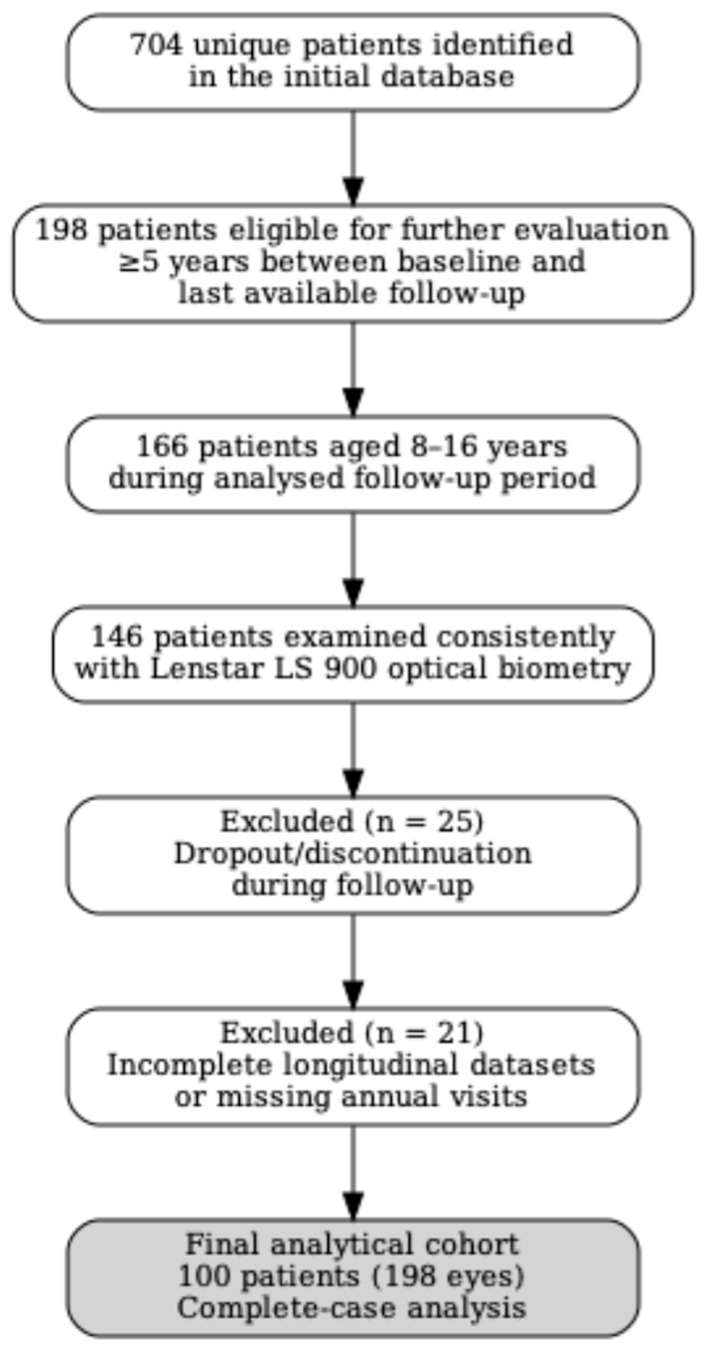
Flow diagram of patient selection, exclusions, and derivation of the final analytical cohort.

The patient selection process was as follows (Figure 1). A total of 704 unique patients were identified in the initial database with the history of orthokeratology fitting. To ensure sufficient longitudinal observation time, only patients with at least 5 years between the baseline examination and the last available follow-up visit were considered eligible for further screening; this determination was based on recorded examination years rather than complete annual visit availability. After applying this criterion, 198 patients remained eligible for further evaluation.

Subsequently, patients aged between 8 and 16 years during the analysed follow-up period were selected based on recorded birth year and examination year, resulting in a cohort of 166 patients, as this age range corresponds to the clinically most active phase of childhood axial elongation and myopia progression.

To reduce measurement variability related to different optical biometers, only patients examined consistently with the same optical biometry method (Lenstar LS 900 optical biometry, Haag-Streit AG) throughout follow-up were included in the final analysis set. This resulted in 146 eligible patients.

Among these patients, 25 were excluded because of dropout or discontinuation during follow-up. The most common reasons included inadequate visual performance, discomfort or intolerance, insufficient overnight lens wear, logistical or financial difficulties, and transition to alternative myopia-control methods.

Additionally, 21 patients had incomplete longitudinal datasets because one or more scheduled examination time points were missing, including missed annual visits during the COVID-19 period. These patients were excluded from the complete-case analysis. No imputation procedures were applied; only participants with complete annual longitudinal datasets were retained in the final analytical cohort (Figure 1).

Consequently, the present study represents a complete-case analysis of patients with available long-term follow-up data. This approach may introduce selection bias and attrition bias and may overestimate treatment-associated outcomes compared with less adherent real-world populations, which should be considered when interpreting the results and generalizability of the findings.

### 2.2. Orthokeratology Lenses and Fitting Procedure

All participants were fitted with Paragon CRT^®^ overnight orthokeratology lenses (Paragon Vision Sciences, Gilbert, AZ, USA). The lenses had a total diameter of 10.5 mm, a central thickness of 0.22 mm, and a 6.0 mm optic zone. The lens material was a fluorosilicone acrylate (Paragon HDS^®^ 100, Paragon Vision Sciences) with a nominal oxygen permeability (Dk) of 127 × 10^−11^ cm^2^/s (mL O_2_/mL·mmHg) and 100 × 10^−11^ cm^2^/s (mL O_2_/mL·mmHg), respectively, depending on lens design. Lenses were selected and fitted by the same experienced practitioner (first author) using a standardised fitting protocol in accordance with the manufacturer’s professional fitting and information guide for Paragon CRT^®^. All clinical follow-up examinations were also performed by the first author.

### 2.3. Biometric Measurements

Axial length and central corneal thickness were measured at baseline and then at least annually using the Lenstar LS 900 (Haag-Streit AG, Köniz, Switzerland), which is based on optical low-coherence interferometry. Measurements were obtained either by the first author or by ophthalmology residents under the supervision and verification of the first author. At each visit, five consecutive measurements were obtained, and the average of these readings was used for analysis. To minimise the impact of diurnal variation in choroidal and axial dimensions [23,24], all measurements were performed between 9:00 a.m. and 1:00 p.m. To avoid overestimating true axial elongation during the initial treatment period, first-year axial length change was interpreted together with the reduction in central corneal thickness (pachymetry).

### 2.4. Statistical Analysis

Axial length was recorded at baseline and at each annual follow-up up to 5 years. To accommodate the bilateral and longitudinal structure of the data, marginal regression models with cluster-robust standard errors clustered on a participant ID were used as the primary approach; this strategy yields valid population-averaged inference without strong assumptions on the random-effect variance structure [25]. The intraclass correlation coefficient for cumulative axial length change was 0.78, justifying the clustered approach. As a pre-specified sensitivity analysis, the primary multivariable model was repeated as a linear mixed-effects model with a random intercept for participant; fixed-effect coefficients and their statistical significance were essentially unchanged. Confidence intervals from cluster-robust estimation reflect population-averaged precision and may appear narrower than corresponding subject-level intervals.

The primary outcome was cumulative axial length change from baseline (dAL) at each follow-up time point. Multivariable models included follow-up year, baseline age at fitting, sex, type of prior optical correction, and baseline spherical equivalent as explanatory variables. Annual AL increments between consecutive intervals (0–1, 1–2, 2–3, 3–4, 4–5 years) were analysed using a linear model with interval as a categorical predictor and cluster-robust standard errors. Because spherical power, cylindrical power, and spherical equivalent are intrinsically collinear, only spherical equivalent was retained as the descriptor of baseline refractive status; variance inflation factors were <2 in the simplified model.

To provide an additional clinically interpretable outcome measure, a responder analysis was also performed. A responder was defined as an eye or participant with an average axial elongation of <0.125 mm/year over the 5-year follow-up period. This threshold was selected to approximate a clinically meaningful reduction relative to the ~0.25 mm/year axial elongation reported in untreated myopic European children [26] of comparable age and is consistent with the documented orthokeratology treatment effect (47–64% reduction in axial progression) [11,12,27]. Responder and non-responder proportions are reported at both eye level and participant level.

A post hoc sample-size calculation assuming a standard deviation of 0.30 mm and a target margin of error of 0.06 mm indicated that approximately 96 eyes were required; the available 198 eyes from 100 children therefore exceeded the minimum requirement.

All statistical analyses were performed using standard statistical software. A *p*-value < 0.05 was considered statistically significant. Results are presented as mean ± standard deviation (SD) unless otherwise indicated.

### 2.5. Ethics

The study was conducted in accordance with the tenets of the Declaration of Helsinki and was approved by the Hungarian National Institute of Pharmacy and Nutrition (OGYÉI/55313/2019). Written informed consent was obtained from all participants; for minors, consent was obtained from a parent or legal guardian.

## 3. Results

### 3.1. Baseline Characteristics

Baseline demographic and clinical characteristics of the study population are summarised in Table 1. The analysis included 100 children undergoing orthokeratology, contributing data from 198 eyes with complete longitudinal biometric follow-up. The cohort comprised 55 boys and 45 girls. The mean age at lens fitting was 11.65 years (range, 8–16 years). Mean baseline spherical equivalent (SE) was −3.39 D (range, −0.75 to −6.00 D), and baseline best-corrected visual acuity was 0.01 ± 0.10 LogMAR. Mean baseline axial length (AL) was 24.70 mm (range, 22.07–26.71 mm), and mean baseline central corneal thickness was 551.7 µm (range, 470–655 µm).

### 3.2. Longitudinal Change in Axial Length

**Figure 2:**
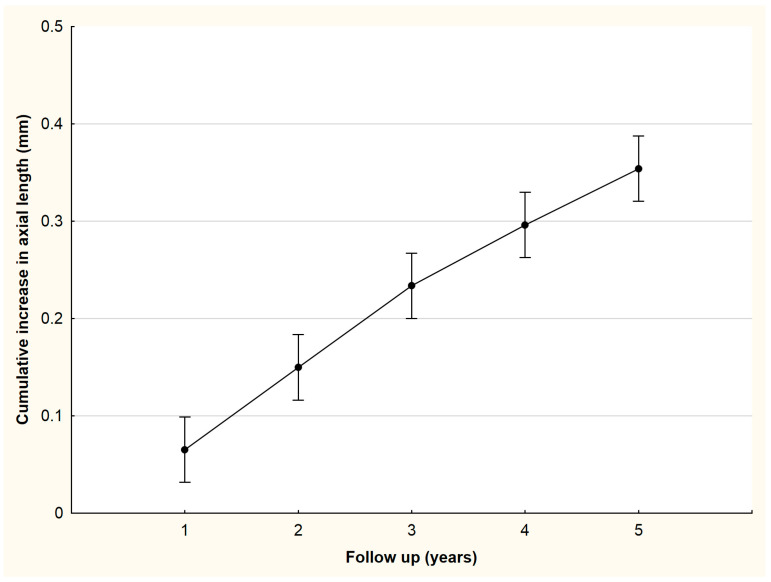
Mean cumulative axial length (AL) change over 5 years of orthokeratology. Mean AL (mm) at baseline and each annual follow-up visit is plotted with 95% confidence intervals. The curve shows a progressive but modest increase in AL over time in children undergoing orthokeratology treatment. Axial elongation was smallest in the first year, increased during years 1–3, and decreased again in years 3–5. A linear model with interval as a categorical predictor and cluster-robust standard errors showed a significant overall difference across the five intervals (global *p* = 0.014).

The following longitudinal analyses reflect the final complete-case cohort with uninterrupted 5-year follow-up and consistent optical biometry measurements and therefore represent a selected subgroup of long-term orthokeratology wearers. Axial length change over time in the overall cohort is shown in Figure 2. Mean AL increased from 24.70 ± 0.79 mm at baseline to 24.75 ± 0.80 mm at year one, 24.83 ± 0.82 mm at year two, 24.92 ± 0.83 mm at year three, 24.98 ± 0.86 mm at year four, and 25.04 ± 0.89 mm at year five. The corresponding mean cumulative AL changes from baseline were 0.048 mm at year one, 0.133 mm at year two, 0.216 mm at year three, 0.279 mm at year four, and 0.337 mm at year five. The annual AL increment varied across the five follow-up intervals. The smallest mean increment was observed during the first year of treatment (0–1 year: 0.048 ± 0.144 mm/year), followed by larger increments during years 1–2 (0.085 ± 0.104 mm/year) and 2–3 (0.084 ± 0.118 mm/year). Thereafter, annual elongation decreased again during years 3–4 (0.063 ± 0.109 mm/year) and 4–5 (0.058 ± 0.088 mm/year). In a linear model with interval as a categorical predictor and cluster-robust standard errors clustered by participant, the overall difference in annual AL increments across intervals was statistically significant (global *p* = 0.014). These findings indicate that axial elongation during orthokeratology was not temporally uniform over the 5-year follow-up period. Importantly, despite this interval-to-interval variability, there was no evidence of progressive acceleration in AL growth over time. Rather, the pattern suggested relatively modest elongation in the first year, somewhat higher growth in the middle years, and lower annual increments again in the later follow-up period.

### 3.3. Multivariable Analysis of Predictors of Axial Length Elongation

To identify factors associated with cumulative axial elongation, a simplified multivariable regression model was fitted using cumulative AL change from baseline (dAL) as the dependent variable and follow-up year, baseline age at fitting, sex, type of prior optical correction, and baseline spherical equivalent as explanatory variables. Standard errors were estimated using a cluster-robust covariance estimator clustered by participant ID. Follow-up year was strongly associated with dAL (β = 0.072 mm/year, 95% CI 0.060 to 0.084, *p* < 0.001), confirming progressive axial elongation over time. Baseline age at fitting was independently associated with cumulative AL elongation (β ≈ −0.05 mm per year of age, *p* < 0.001), indicating that younger age at treatment initiation was associated with greater AL increase over time. Baseline spherical equivalent was not significantly associated with dAL (*p* = 0.122) (Table 2). Sex and prior optical correction were not significantly associated with cumulative AL elongation in the simplified model (all *p* > 0.5). A linear mixed-effects model with a random intercept for participant yielded essentially identical fixed-effect estimates.

### 3.4. Relationship Between Baseline Age and Axial Length Change

**Figure 3:**
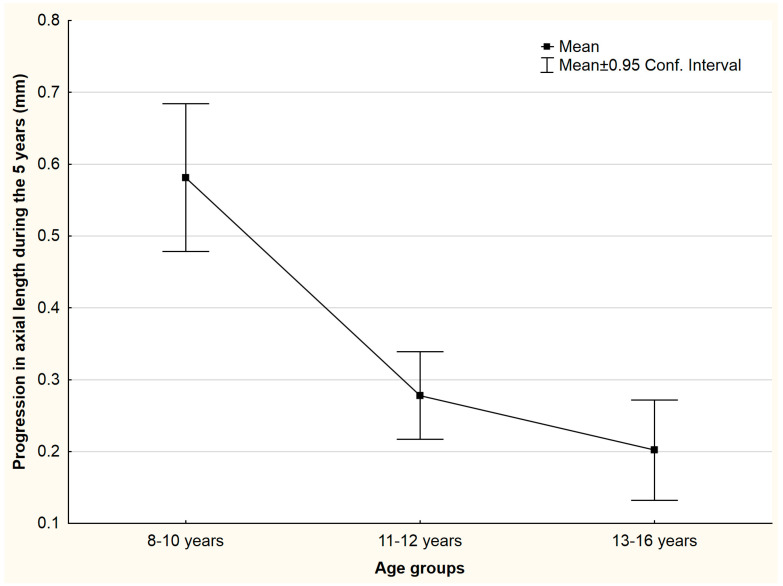
Five-year axial length progression by age group at orthokeratology initiation. Mean cumulative axial length change (mm) over 5 years is plotted for three baseline age groups (8–10, 11–12, and 13–16 years), with 95% confidence intervals shown as error bars. Younger children demonstrated greater axial elongation over the 5-year period than older children.

To further illustrate the strong age dependence of treatment-associated axial elongation, the association between baseline age and 5-year axial length change was examined at the eye level (Figure 3). The mean 5-year axial elongation decreased progressively from approximately 0.60 mm in children aged 8–10 years to 0.30 mm in those aged 11–12 years and 0.20 mm in those aged 13–16 years, with a significant overall difference across age groups (*p* < 0.01). Consistent with the multivariable analysis, a significant negative trend was observed between age at fitting and cumulative AL elongation, indicating that younger children at orthokeratology initiation experienced greater axial elongation over the 5-year follow-up, whereas older children showed smaller cumulative changes. The graphical visualisation highlights the clinically relevant influence of baseline age on long-term axial elongation during orthokeratology treatment.

**Figure 4:**
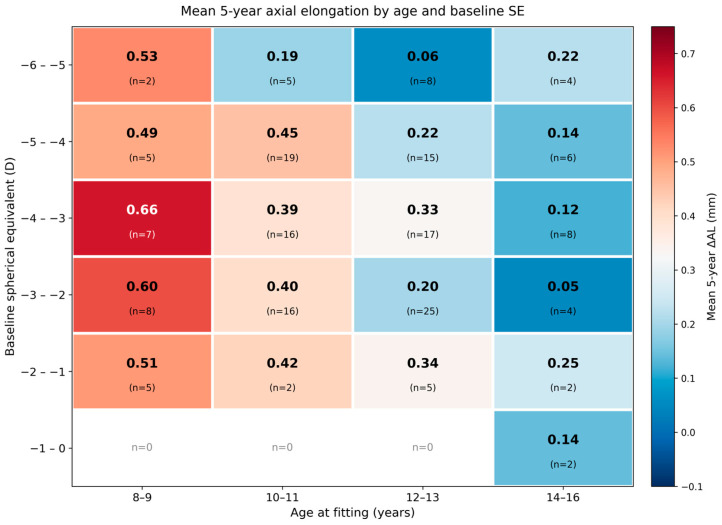
Heatmap of mean 5-year axial elongation by age and baseline spherical equivalent. Each cell represents the mean 5-year axial length (AL) change for eyes grouped by age at orthokeratology initiation (*x*-axis, 2-year bins) and baseline spherical equivalent (*y*-axis, 1.0 D bins). Colours indicate the magnitude of mean AL change (mm), with cooler colours representing smaller elongation and warmer colours representing greater elongation. The heatmap illustrates that older children with higher baseline myopia tended to exhibit the lowest axial elongation over the 5-year follow-up, whereas younger children with moderate baseline myopia generally showed higher elongation. Cell annotations indicate the number of eyes contributing to each category combination.

To further characterise the joint influence of age and baseline myopia at the eye level, an additional regression model was fitted with 5-year axial length change as the dependent variable and baseline age and spherical equivalent as fixed effects, using subject-level clustering to account for correlation between fellow eyes. In this more parsimonious age–SE model with clustered standard errors, baseline age remained significantly associated with 5-year AL change (β ≈ −0.09 mm per year, *p* < 0.01), and the independent contribution of baseline SE was weaker and did not reach statistical significance after adjustment. To illustrate these relationships, Figure 4 displays a heatmap of mean 5-year AL change across 2-year age bins and 1.0 D baseline SE bins. The figure suggests a gradient in treatment response, with the lowest mean axial elongation clustering in older age groups with higher baseline myopia and higher elongation values concentrated among younger children with lower baseline myopia, mirroring the associations detected in the clustered age–SE model.

### 3.5. Early Axial Length Change as a Predictor of Long-Term Outcome

Pearson correlation analysis was used to assess the association between first-year and 5-year axial length change, as well as between 3-year and 5-year axial length change. First-year axial elongation was strongly associated with the cumulative 5-year axial length change (r = 0.74, *p* < 0.001). However, because first-year AL change contributes algebraically to cumulative 5-year elongation, this estimate is partly inflated by mathematical coupling. When the analysis was repeated using the AL change between years 1 and 5 (i.e., excluding the first-year contribution), the correlation with first-year AL change decreased to r = 0.41 (*p* < 0.001), suggesting that early treatment response may still provide clinically useful prognostic information regarding subsequent axial elongation, although the association is more modest after removal of the mathematical overlap between the two measures. An even stronger association was observed between the 3-year and 5-year axial length changes (r = 0.90, *p* < 0.001).

### 3.6. Responder Analysis

To provide an additional clinically interpretable outcome, a responder analysis was performed. Using the biologically anchored threshold of <0.125 mm/year, 169 of 198 eyes (85.4%) were classified as responders, while 29 eyes (14.6%) were classified as non-responders. At the participant level, 83 of 100 children (83.0%) were classified as responders and 17 (17.0%) as non-responders. To explore potential predictors of responder status, a subject-level logistic regression model was fitted with responder status as the dependent variable and baseline age and baseline spherical equivalent as predictors. In this exploratory model, neither baseline age (OR 1.81, 95% CI 0.89 to 3.71, *p* = 0.103) nor baseline spherical equivalent (OR 1.93, 95% CI 0.57 to 6.59, *p* = 0.293) showed a statistically significant independent association with responder status. These responder proportions should be interpreted in the context of the selected complete-case cohort with uninterrupted long-term follow-up and may overestimate responder rates in less adherent real-world populations.

### 3.7. Safety Outcomes

Importantly, no cases of microbial keratitis were observed in this cohort during the follow-up period. However, the present study was not designed or powered to evaluate the safety profile of orthokeratology or the incidence of rare adverse events, which have been addressed separately in our previously published safety analysis [28].

## 4. Discussion

Myopia is an increasingly important public health challenge, and recent consensus statements support its recognition as a disease requiring active management [4]. Within this landscape, orthokeratology is regarded in Europe as one of the most effective stand-alone optical strategies for myopia control, although treatment effects vary across studies and lens designs [8,12]. The present study adds long-term real-world evidence from a selected Hungarian paediatric cohort with 5 years of follow-up. In this cohort, mean cumulative axial length change reached approximately 0.35 mm at 5 years, corresponding to an annualised elongation of about 0.07 mm/year, which appeared to fall at the lower end of the range reported in previous European orthokeratology cohorts (Table 3).

### 4.1. Comparison with European Studies

To place the present findings in context, AL outcomes were compared with prior European orthokeratology studies. This comparison is important because most long-term orthokeratology data in White or European children derive from relatively small cohorts, shorter observation periods, or both.

When viewed descriptively alongside previously published European orthokeratology cohorts, the annualised axial elongation observed in the present study appears numerically lower than most prior reports. However, these comparisons should be interpreted cautiously because of substantial differences in study design, sample selection, age distribution, ethnicity, lens types, and follow-up duration across studies. The lower annual elongation observed in our highly selected cohort is therefore consistent with but does not prove a particularly favourable axial length outcome relative to other European orthokeratology populations. The requirement for uninterrupted 5-year follow-up with consistent biometric measurements likely enriched the cohort for highly adherent long-term orthokeratology wearers and motivated families with stable follow-up behaviour. As a result, the present cohort may represent a best-case real-world subgroup, which could partly explain the particularly favourable long-term outcomes observed. Accordingly, the low annual axial elongation and high responder proportions observed in the present study should be interpreted in the context of regular long-term follow-up and may not be directly generalisable to all orthokeratology-treated children. The favourable long-term outcome is also compatible with previous reports suggesting that orthokeratology can maintain myopia-control efficacy over several years without clear evidence of treatment fatigue [18,20].

### 4.2. Historical Controls

To interpret the magnitude of AL elongation against expected ocular growth, 1- and 2-year outcomes were compared with historical emmetropic and myopic cohorts from the Orinda Longitudinal Study of Myopia (OLSM) [6,7] and the Collaborative Longitudinal Evaluation of Ethnicity and Refractive Error (CLEERE) Study [32]. Although these are indirect comparisons, they provide a useful clinical benchmark (Table 4).

In this framework, axial elongation in the present cohort was lower than the values reported in historical myopic controls and approached the range reported in historical emmetropic cohorts. However, because emmetropic and myopic eyes follow different growth trajectories, these comparisons should be interpreted only as indirect clinical benchmarks rather than as evidence of physiological normalisation of axial growth. This does not prove efficacy in the same sense as a randomised controlled trial, but it strengthens the biological plausibility that orthokeratology substantially slowed axial growth in this population.

### 4.3. Population Context

Although the favourable outcomes observed in Hungarian children undergoing orthokeratology might raise the possibility that myopia progression is intrinsically slower in this population, available epidemiological data do not support this assumption. Historical data from Hungary show that myopia prevalence among young adults (18–35 years) increased from approximately 7% in earlier decades to 58.7% in recent national surveys, indicating a substantial generational shift [33]. European comparisons also do not suggest a uniquely protected population, as Hungary ranks among countries with relatively high reported myopia prevalence [2]. Therefore, the favourable axial length outcomes observed in the present cohort are unlikely to be explained solely by population-level epidemiological differences.

### 4.4. Predictors and Timing

Baseline age was one of the strongest predictors of axial length progression in this cohort. Younger age at treatment initiation was consistently associated with greater cumulative elongation, whereas older children showed smaller long-term AL changes, in agreement with both the multivariable modelling and the age-stratified analyses. This age-dependent pattern is biologically plausible because younger eyes remain in a more active growth phase with greater responsiveness to visual signals regulating ocular growth. This interpretation is concordant with previous evidence showing that younger children exhibit faster axial growth and a higher risk of progressive myopia even during active treatment [5,6]. Clinically, these findings suggest that orthokeratology is effective across the paediatric age range, but that younger patients should be considered a higher-risk subgroup and monitored more closely.

Beyond baseline age, early treatment response may also provide important prognostic information. Previous studies have shown that early axial length changes during orthokeratology treatment may predict long-term myopia progression [16,34]. A similar pattern was observed in the present cohort, where first-year axial elongation was associated with cumulative 5-year axial length change. Although part of this association reflects mathematical coupling between early and cumulative measures, the relationship remained statistically significant even after exclusion of the first-year contribution, supporting the clinical usefulness of early treatment response as a prognostic indicator that may help identify children who require closer monitoring or earlier treatment adjustment.

Recent evidence also suggests that suboptimal responders to orthokeratology monotherapy may benefit from adjunctive pharmacological treatment, particularly low-dose atropine [35]. Since first-year axial elongation was strongly predictive of long-term outcome in the present cohort, this metric may serve as an early indicator for identifying children who could require combination therapy to achieve optimal myopia control.

Baseline refractive status showed no significant linear association with axial length (AL) elongation in the multivariable models in this study, in contrast to previous reports suggesting that higher baseline myopia is linked to slower axial elongation during orthokeratology treatment [13,14]. Notably, heatmap-based subgroup analysis revealed a more nuanced pattern, with younger children exhibiting mild myopia (−2 to −4 D) demonstrating the greatest axial elongation. These findings suggest that the relationship between baseline spherical equivalent and treatment response may be non-linear and age-dependent. Prior hypotheses have attributed reduced axial elongation in higher myopia to stronger treatment-induced optical effects, such as increased higher-order aberrations and enhanced peripheral retinal defocus; however, such effects were not evident as a linear trend in the present cohort [15,36,37]. While conventional analyses did not demonstrate a clear association, heatmap-based exploration suggested a more complex, non-linear pattern, with younger children in the mild myopia range (−2 to −4 D) exhibiting the greatest axial elongation despite treatment. These findings indicate that treatment response is not driven by baseline spherical equivalent as an independent linear predictor but may instead reflect an interaction between age and refractive status. These findings underscore the importance of lens customization, particularly in eyes with moderate baseline refractive error. In such cases, optimising lens design to achieve sufficient corneal reshaping may be necessary to induce a stronger relative myopic shift in the peripheral retina and thereby enhance the myopia control effect, consistent with recent evidence supporting increased compression factor designs [14,37].

By contrast, sex and prior optical correction did not emerge as significant predictors in the combined 5-year analyses, implying that the long-term impact of orthokeratology was driven primarily by biological growth stage and baseline refractive characteristics rather than by sex or correction history.

### 4.5. Temporal Pattern

Treatment effect was not temporally uniform across follow-up. Mean cumulative AL change was approximately 0.048 mm at year 1, 0.133 mm at year 2, 0.216 mm at year 3, 0.279 mm at year 4, and 0.337 mm at year 5, while interval-specific annual growth differed significantly across time (global *p* = 0.014). The largest annual increments were observed in the middle follow-up period, whereas the first and last intervals showed smaller growth rates. This pattern may reflect the combined influence of age-related physiological slowing of axial growth and variation in treatment-associated control effects across different stages of follow-up. The relatively small elongation observed during the first year is also consistent with the early treatment effect commonly reported in orthokeratology studies [10,22]. One possible explanation for the particularly small axial elongation observed during the first year of treatment is transient orthokeratology-associated choroidal thickening, which may temporarily reduce measured axial length and may exert a stronger effect during the initial phase of treatment, as suggested by previous reports [23,24].

Overall, these findings argue against a simple assumption that treatment efficacy remains identical year after year, but they also do not support progressive loss of effect; rather, orthokeratology appeared to maintain overall control of axial elongation across the full 5-year observation window.

### 4.6. Strengths and Limitations

The main strengths of this study are the large real-world cohort, the 5-year follow-up, and the availability of repeated annual axial length measurements obtained with the same instrument. In addition, the inclusion of both eyes with participant-level clustering allowed use of the full dataset while appropriately accounting for within-subject correlation.

The principal limitation is the absence of a concurrent untreated single-vision spectacle or contact lens control group, which precludes definitive causal inference. Comparisons with previously published European orthokeratology cohorts also remain indirect and should be interpreted cautiously because of substantial heterogeneity between studies. In addition, the favourable outcomes observed here should therefore be interpreted in the context of a selected population of compliant long-term wearers. The final analytical dataset also represents a complete-case analysis restricted to patients with uninterrupted long-term follow-up, complete annual biometric measurements and AL measurement with the same device. This approach ensured a more uniform longitudinal dataset and avoided varying sample composition across the 5-year follow-up period. Among the 146 patients eligible after age-based selection and restriction to uniform optical biometry, 46 patients (31.5%) were excluded because of treatment discontinuation or incomplete longitudinal follow-up, including missed visits during the COVID-19 period. Although attrition is expected in long-term real-world paediatric follow-up studies and has also been observed in previous long-term European orthokeratology cohorts [18], the final analytical cohort may nevertheless have favoured patients with more consistent follow-up behaviour and treatment adherence. Consequently, the favourable long-term axial length outcomes observed in this study may partly reflect patient selection in addition to treatment-associated effects. Further limitations include the retrospective design, the lack of systematically collected data on parental myopia and lifestyle-related factors such as outdoor activity and near-work exposure, and the inability to assess some potentially relevant optical factors, such as lens decentration, in a standardised manner across the full study period.

Confidence intervals derived from eye-level analyses with cluster-robust standard errors yield population-averaged precision rather than direct biological precision and may therefore appear narrow compared with subject-level estimates.

## 5. Conclusions

Orthokeratology in this large, real-world Hungarian cohort was associated with low axial elongation over 5 years, with a mean cumulative increase of only about 0.35 mm (≈0.07 mm/year). This annual rate appears to fall at the lower end of the range reported in previous European orthokeratology cohorts, although indirect comparisons between studies should be interpreted cautiously because of differences in study design and patient populations. Under the biologically anchored responder definition of <0.125 mm/year, 85.4% of eyes and 83.0% of participants were classified as responders, supporting favourable long-term myopia-control outcomes in this setting.

Analyses also identified clinically relevant predictors within this favourable overall outcome. Younger baseline age was consistently associated with greater axial elongation, whereas children who initiated orthokeratology at an older age and with higher baseline myopia tended to show the smallest 5-year AL changes. In contrast, sex and prior optical correction did not exert a meaningful independent effect in the long-term models. Early axial length changes also proved clinically informative, as first-year elongation remained associated with subsequent long-term outcome even after accounting for the effect of cumulative measurements. These findings emphasise that, even in a cohort with generally excellent control, age, baseline refractive status, and early treatment response all contribute to individual treatment outcomes.

Although the retrospective design, the absence of a concurrent control group, and the selection bias inherent in the inclusion criteria limit definitive causal inference, our findings should be interpreted as descriptive treatment-associated outcomes within a highly selected cohort rather than definitive evidence of treatment efficacy. The convergence of our results—low annual elongation, strong age dependence, and high responder rates—with the broader orthokeratology literature nevertheless supports their clinical plausibility. Overall, our data suggest that, under routine European practice conditions, well-fitted orthokeratology may be associated with sustained low axial elongation in carefully selected and adherent myopic children.

## Figures and Tables

**Table 1:** Baseline demographic and clinical characteristics of the study population.

Parameter	Value
Patients/Eyes	100/198
Male/Female	55/45
Age at fitting	11.65 years (8–16 years)
Baseline spherical equivalent (SE)	−3.39 D (−0.75 to −6.0 D)
Baseline best corrected visual acuity (LogMAR)	0.01 ± 0.10
Baseline axial length	24.70 mm (22.07–26.71 mm)
Baseline pachymetry	551.7 µm (470–655)

**Table 2:** Multivariable regression model for cumulative axial length change (dAL, mm) over 5 years of orthokeratology, with cluster-robust standard errors clustered by participant ID.

Predictor	β (mm)	95% CI	*p*-Value
Follow-up year (per 1 year)	0.071	0.059 to 0.083	<0.001
Age at fitting (per 1 year)	−0.049	−0.070 to −0.029	<0.001
Sex (F vs. M)	0.036	−0.043 to 0.115	0.374
Prior correction: spectacles vs. none	−0.061	−0.196 to 0.074	0.373
Prior correction: contact lenses vs. none	−0.038	−0.200 to 0.125	0.650
Spherical equivalent (per 1 D)	0.027	–0.007 to 0.061	0.122

**Table 3:** Axial length changes reported in European orthokeratology studies and in the present study.

Author(s)	Year	Patient’s Ethnicity	Age (Years)	Follow-Up	No. of Patients	AL Measurement	AL Changes (mm)
Santodomingo-Rubido [29]	2012	White European	6–12	2 years	29	Optical biometry	0.47 ± 0.18
Santodomingo-Rubido [18]	2017	White European	6–12	7 years	14	Optical biometry	0.91 ± 0.31
Pauné [30]	2015	Caucasian	9–16	2 years	18	Ultrasound	0.32 ± 0.20
Queirós [31]	2024	French	7–17	1 year	25	Optical biometry	0.06 ± 0.13
Jakobsen [19]	2025	Scandinavian	6–12	2 years	15	Optical biometry	0.29
Jakobsen [19]	2025	Scandinavian	6–12	3 years	14	Optical biometry	0.48
This study	2026	Hungarian	8–16	1 year	100	Optical biometry	0.07 ± 0.14
This study	2026	Hungarian	8–16	2 years	100	Optical biometry	0.15 ± 0.20
This study	2026	Hungarian	8–16	3 years	100	Optical biometry	0.23 ± 0.26
This study	2026	Hungarian	8–16	5 years	100	Optical biometry	0.35 ± 0.34

AL = axial length.

**Table 4:** Comparison of 1- and 2-year axial elongation in orthokeratology-treated myopic children and historical control cohorts.

Follow-Up	OLSM Emmetropic Caucasian 6–14 Years	OLSM Myopic Caucasian 6–14 Years	CLEERE Emmetropic USA 6–14 Years	CLEERE Myopic USA 6–14 Years	This Study OK-Treated Hungarian 8–16 Years
1 year	0.10 mm	0.25 mm	0.10 mm	0.17 mm	0.05 mm
2 years	0.18 mm	0.48 mm	0.20 mm	0.34 mm	0.16 mm

(CLEERE myopic annual axial elongation refers to post-onset years; Mutti et al. reported post-onset annual elongation in the range of approximately 0.10–0.17 mm/year; the value of 0.17 mm/year represents the upper bound of the reported range; two-year CLEERE values were calculated as twice the annual rates because cumulative 2-year axial elongation was not directly reported in CLEERE publications) [32]. OLSM = Orinda Longitudinal Study of Myopia; CLEERE = Collaborative Longitudinal Evaluation of Ethnicity and Refractive Error; OK = orthokeratology; AL = axial length.

## Data Availability

The data presented in this study are not publicly available due to privacy and institutional restrictions but are available from the corresponding author upon reasonable request.

## Abbreviations

The following abbreviations are used in this manuscript:

AL	Axial Length
dAL	Cumulative axial length change from baseline
SD	Standard deviation
SE	Spherical equivalent
OGYÉI	National Institute of Pharmacy and Nutrition, Hungary
logMAR	Logarithm of the minimum angle of resolution
F	Female
M	Male
D	Diopter
CI	Confidence interval
OLSM	Orinda Longitudinal Study of Myopia
CLEERE	Collaborative Longitudinal Evaluation of Ethnicity and Refractive Error
OK	Orthokeratology
